# Supplementary material for: Are carbon emissions trading and green financial instruments synergistic? -Comprehensive quantitative research based on content analysis
Source: PLoS One. 2024 Mar 7;19(3):e0298601. doi: 10.1371/journal.pone.0298601 (PMC10919601; doi:10.1371/journal.pone.0298601)
Supplement: S1 Data — (ZIP) [file pone.0298601.s001.zip › Data/地级市Z_F工作报告.zip!/╘╞─╧/╙±╧¬╩╨/└┤╫╘╣1⁄2╓┌║┼í╛┬φ┐╦╩2╛▌═°í┐_2015.docx]

我代表市人民政府，向大会报告政府工作，请各位代表审议，请市政协各位委员提出意见。

　　一、2014年工作回顾

　　过去一年，在市委坚强领导下，市人民政府围绕市四届人大二次会议确定的目标任务，以党的、十八届三中、四中全会和系列重要讲话精神为指导，集中精力打好产业建设年、改革创新年、基础设施建设年和生态文明建设年攻坚战，克服市场需求持续低迷、经济下行压力增大、畜禽疫病高发等困难，经济保持平稳发展，各项工作成效明显。全市完成生产总值1184.7亿元、增长8%，地方公共财政预算收入113.6亿元、增长7.2%，规模以上固定资产投资511.9亿元、增长30%，社会消费品零售总额255.6亿元、增长13%，城镇居民人均可支配收入27223元、增长10.1%，农村居民人均可支配收入9969元、增长13.2%，城镇化率45.1%，居民消费价格总水平上涨2.1%，城镇登记失业率3.3%，人口自然增长率5.3‰，万元生产总值能耗下降5.7%。

　　我们重点抓了6个方面的工作：

　　第一，努力保持经济平稳增长。落实省政府16条、制定市政府15条稳增长措施，强化运行分析和专项督查，千方百计稳增长。抓好农业稳增长。编制高原特色农业发展规划，制定发展现代农业庄园、加快畜牧业可持续发展等意见，扶持重点产业，培育新型经营主体。完成烤烟生产收购任务，蔬菜、水果、核桃等特色产业有了新发展，种植业产值120.9亿元，增长4.6%。妥善处置畜禽疫病，稳定畜禽养殖规模，畜牧业产值79.1亿元，增长7.7%。推动农业适度规模经营，新增土地流转面积3.4万亩，启动18个农业庄园建设，褚橙庄园、玉溪庄园等8个省级精品庄园带动能力和影响力不断扩大，认定50个家庭示范农场，新增15户龙头企业、71个专业合作社，农业增加值达122.8亿元，增长6%。突出工业稳增长。制定工业转型升级、化解产能过剩实施方案，强化要素保障，及时解决企业生产经营中的困难问题。卷烟及配套产业增加值386.5亿元、增长8.1%，矿冶业增加值137.5亿元，装备制造、生物医药及食品、新能源新材料、信息等新兴产业快速发展。华为云计算数据中心、韵雅黄腐酸等一批重大项目顺利推进，5000万元以上重点项目竣工投产38个，新增规模以上企业33户。加快园区建设发展，成立4个土地储备分中心，收储土地1.56万亩，基础设施建设投入20.1亿元，建成标准厂房50万平方米，工业园区完成增加值590.3亿元，增长8.7%。制定加快民营经济发展实施意见和考核办法，实现增加值402亿元，增长9.1%。工业增加值670.4亿元，增长8.1%。扩大消费稳增长。出台加快旅游产业发展的决定，澄江寒武纪乐园、太阳山国际生态旅游度假区等21个重大旅游项目稳步推进，旅游总收入108.6亿元，增长26.9%。推进国家信息消费试点城市工作，启动信息惠民、宽带乡村示范工程。加强专业市场建设，完成4个乡镇农贸市场改造，建成红塔区精品建材市场。优化外贸结构，农产品出口增势强劲，进出口总额9.7亿美元，增长35.7%。第三产业增加值355.5亿元，增长7.4%。增加投资稳增长。坚持完善重大项目市级领导联系制度，组织重点项目集中开工，101个亿元以上重点项目完成投资248.7亿元。加强基础设施建设，昆玉铁路扩能改造和晋红、呈澄、石红高速公路等项目进展顺利，建成农村公路350公里;改造中低产田地19万亩，完成水源工程5件、小(二)型病险水库除险加固40件，库坝蓄水5.3亿方;1个220千伏、3个110千伏输变电工程竣工，戛洒江一级电站和羊岔街、向阳山风电场开工。创新融资方式，发行企业债券19亿元，争取中央代云南发行地方政府债券转贷资金6.5亿元，金融机构新增贷款68.7亿元，68户企业在前海股权交易中心挂牌，万绿生物成功在新三板上市。争取上级资金114.6亿元。完善招商引资考核办法，开展“百名浙商玉溪行”等推介活动，引进市外到位资金605.4亿元、增长49%，利用外资7430万美元、增长10%。

　　第二，稳步推进重点领域改革。推进行政审批制度改革，取消市级审批项目25项，下放20项，调整合并拆分47项。工商登记制度改革成效显著，新增注册企业增长2倍。制定园区实体化管理指导意见，启动高新区改革试点。“营改增”试点顺利推进。市商业银行增资扩股2.2亿元，7个市属投融资公司实际融资216亿元。完成乱埋乱葬清理整治任务，6个县区提前实现殡葬改革“两个100%”目标，全市火化率达49.8%。农村土地承包经营权确权登记颁证、集体林权制度、土地征收补偿费作价入股、水务、供销社等改革有序推进。改革高中招生录取办法，公立医院改革取得成效，生态文明、文化等体制改革稳步推进。

　　第三，统筹推进城乡协调发展。出台推进玉溪新型城镇化发展的实施意见，完成玉溪发展战略规划、中心城区城市设计和公共设施、绿道网络等专项规划编制，完成5个县城总规修编和滇中民居设计，新平列入全国县域乡村建设规划工作试点。加快中心城区建设，平战结合人防工程、排水管网改扩建一期、高仓立交改扩建、东近面山绿化等工程完工，新天地商业广场、红星国际广场城市综合体建设有序推进。城南客运站开工建设，建成公交站台188个、站点538个。铺设燃气管道47.3公里，推广居民用户2万户。制定城市精细化管理实施方案，持续抓好拆临拆违，拆后绿化43万平方米。实施美丽乡镇三年行动计划和美丽乡村建设行动计划，20个美丽乡镇、101个美丽乡村建设有序推进。开展城乡垃圾整治行动，建成污水收集管网48.3公里，重点集镇“一水两污”项目开工8个。成功申报6个国家级传统文化村落。新增“农转城”9.4万人，转户居民参加城镇社保15.5万人次。

　　第四，加强生态环境保护。制定“三湖”四县生态建设考核办法、抚仙湖保护管理实施办法，扎实推进“三湖两库”保护治理，“三湖”水污染综合防治“十二五”规划项目开工率98.5%、完工率63.6%，抚仙湖总体水质稳定保持Ⅰ类，星云湖、杞麓湖水质下降趋势得到遏制。抓好抚仙湖良好湖泊生态环境保护试点，争取上级资金6.7亿元，北岸生态湿地项目搬迁群众1500人、建成湿地631亩，调整种植结构，发展蓝莓、荷藕等低污染作物1.3万亩。争取星云湖保护治理专项资金1亿元，杞麓湖列入国家湿地公园建设试点。东片区暨“三湖”生态保护水资源配置应急工程有序推进，开始分段试压。东风水库、飞井海水库和16条河道综合整治成效明显。实施绿化造林41.4万亩，治理水土流失196平方公里，新增耕地1.6万亩。强化工业园区、大型施工场地污染综合治理，建成12条机动车环保检测线，淘汰黄标车及老旧车1.6万辆，启动8个县城环境空气质量监测，中心城区空气质量一级天数增加63天。落实区域限批整改措施，完成减排项目150个，淘汰117万吨钢铁、28.6万吨水泥落后产能。

　　第五，千方百计保障改善民生。加大民生投入，支出占公共预算支出的75%，10件惠民实事圆满完成。新增城镇就业2.2万人，城镇失业、就业困难人员再就业1.1万人，扶持自主创业1.2万人，培训农村劳动力2.8万人、转移2.7万人。城乡居民养老保险并轨运行，提高最低生活保障补助标准、惠及13.6万人，企业退休人员养老金提高10%。城镇职工医保最高支付限额达25万元，新农合人均筹资标准提高到435元。完成1个整乡推进、206个整村推进扶贫项目，5万人实现脱贫。建成“爱心水窖”1.2万口、农村饮水安全工程277件，解决了8.6万人饮水安全问题。建成保障性住房1.45万套，改造农村危房1.1万户，安置溪洛渡水电站外迁化念移民2997人。完成10个农村敬老院、50个居家养老服务中心、103个农村幸福院、102个老年活动室建设。加快美丽100校园行动计划暨校舍安全工程建设，加固改造39.5万平方米，拆除重建17.6万平方米。在全省率先启动教育信息化建设，民办中小学实现同城同教。学前教育毛入园率达93%。推行学区化办学，试行优秀教师多点执教，高中办学质量跻身全省前列。《聂耳》、《澄江化石地》、《神奇抚仙湖》中小学课外读物纳入全国素质教育基础工程教材。制定创新型玉溪建设政策措施，启动科技特派员创新创业行动，举办生物资源开发论坛和中国院士玉溪行活动，新增2个院士工作站、4个工程技术研究中心、1个重点实验室、11户国家高新技术企业。县乡村医疗服务一体化管理基本全覆盖，完成6个乡镇卫生院、31个村卫生室标准化建设。市中医院外科大楼建成使用，引进社会资本建设西南国际医院暨健康产业园、参与市儿童医院建设管理。启动实施“单独两孩”政策。澄江化石地保护列入省人大立法计划，化石博物馆开工建设。实施文化站改扩建30个、文化惠民示范村项目6个，滇剧《水莽草》荣获“五个一工程”奖，完成地方节目无线覆盖一期工程。承办网球、自行车、帆船等国内外赛事，参加第十四届省运会取得好成绩。加强基层社会治理，5个社区命名为全国示范社区。依法管理宗教事务，民族团结进步边疆繁荣稳定新平示范县、盘溪示范镇建设取得实效。加强信访工作，市县乡三级视频接访网络建设有序推进。出台加强反恐维稳工作意见，平安玉溪建设深入推进，社会保持和谐稳定。开展安全生产专项整治，加强食品药品监管，安全生产形势稳定。妇女儿童、老龄、残疾人、红十字、慈善、关心下一代等事业健康发展，外事侨务、防灾减灾、统计、人防、双拥、史志等工作取得新成绩。

　　第六，切实抓好政府自身建设。制定加强司法行政促进依法治市实施意见，“四五”依法治市和“六五”普法有序推进。深入开展党的群众路线教育实践活动，着力整治“四风”，干部作风明显好转，服务质量不断提高。坚持党的领导，自觉接受人大法律监督、工作监督和政协民主监督，人大代表建议和政协委员提案办结率均达100%，解决率分别为45.5%、44.3%。组织开展集体学法，重大决策听证65次、社会公示4968次、重点工作通报9443项，科学民主依法决策能力不断提高。电子政务协同办公系统全面推开，行政审批网上政务服务大厅建设全省领先，政府投资建设项目中介库建成运行。完成“吃空饷”专项清理工作，制定市级机关差旅费管理办法、党政机关国内公务接待管理实施细则等规章制度，“三公”经费支出下降29%。加大审计监察力度，强化督促检查，行政问责148人。落实党风廉政建设主体责任，纠风治乱成效明显。

　　各位代表，回顾去年的工作，成绩来之不易。这是市委坚强领导，市人大、市政协监督支持，全市人民共同努力的结果。在此，我代表市人民政府，向全市广大干部群众，向人大代表、政协委员，向各民主党派、工商联、各人民团体和社会各界人士，向中央和省驻玉单位、军警部队官兵，向所有关心支持玉溪发展的同志们、朋友们，表示崇高的敬意和衷心的感谢!

　　各位代表，总结去年的工作，虽然大部分经济指标超过全省平均水平，但生产总值、财政收入、固定资产投资等增速仍低于年初确定目标。这既受宏观经济下行、工业产品价格下滑、生产要素成本上升、房地产市场调整等客观因素影响，也暴露出我们对新常态下经济增速放缓估计不足，一些部门行政效率不高，不作为、选择性作为，稳增长、调结构、促改革措施落实不到位等主观方面的问题。综合分析判断，当前经济社会发展仍面临不少困难和问题：一是产业培育发展滞后，支柱产业单一，发展方式粗放、质量效益不高，转型升级任务重。二是企业生产经营困难，税收增长放缓，偿债压力加大，财政收支矛盾突出，保运转、保稳定、保民生难度增加。三是大项目好项目储备不足，招商环境不优，融资难、融资贵等问题突出，重大项目推进缓慢，投资增长乏力。四是资源环境约束加剧，水体、大气、土壤等环境污染形势严峻，节能减排和化解过剩产能压力大。五是城乡协调发展任务重，社会管理方式有待创新，土地征用、房屋拆迁等热点难点问题仍然突出。解决好这些困难和问题，促进经济社会持续健康发展，我们必须保持定力，攻坚克难，扎实工作，努力创造新业绩，向全市人民交上一份合格答卷。

　　二、2015年目标任务

　　今年是全面深化改革的关键之年，是全面推进依法治市的开局之年，也是全面完成“十二五”规划的收官之年。政府工作的总体要求是：坚持以党的十八届三中、四中全会、中央经济工作会精神为指导，全面贯彻系列重要讲话和视察云南时的重要指示，认真落实省委、省政府和市委的决策部署，坚持稳中求进的总基调，坚持以提高经济发展质量和效益为中心，坚定不移地实施生态立市、农业稳市、工业强市、两烟富市、文化旅游兴市战略，把转方式调结构放到更加重要的位置，狠抓改革攻坚，突出创新驱动，加强民生保障，强化风险防控，加快建设美丽玉溪，努力干在实处，走在前列，促进玉溪经济平稳健康发展和社会稳定。全市经济社会发展主要目标建议为：生产总值增长10%左右，地方公共财政预算收入增长7%左右，规模以上固定资产投资增长28%左右，社会消费品零售总额增长13%以上，城镇居民家庭人均可支配收入增长11%以上，农村居民家庭人均可支配收入增长13%以上，城镇化率提高2个百分点，居民消费价格指数控制在103%以内，城镇登记失业率控制在4.3%以内，人口自然增长率控制在5.8‰以内，万元生产总值能耗下降2%以上。

　　做好今年的工作，我们必须深刻认识、准确把握新常态的基本特征。我国经济发展进入新常态后，发展速度由高速转向中高速，发展方式从规模速度型粗放增长转向质量效率型集约增长，结构从增量扩能为主转向调整存量、做优增量并存的深度调整，发展动力从传统增长点转向新的增长点。新常态下，玉溪经济发展靠拼劳力、拼资源实现高速增长已经难以为继，靠模仿型、排浪式消费拉动经济增长的历史已经基本结束，靠低成本、低附加值初级产品净流出拉动经济增长的状况亟待改善。一定要认识新常态，适应新常态，激发新动力，以改革创新精神谋划发展、推动工作。我们必须深刻认识、准确把握新常态下面临的重大机遇。当前，世界经济处在国际金融危机后的深度调整期，经济增速预计略有回升，但总体仍然疲弱;我国正处在增长速度换档期、结构调整阵痛期、前期刺激政策消化期“三期叠加”阶段，经济下行压力加大。同时也要看到，国家实施“一带一路”、长江经济带等战略，我省建设全国民族团结进步示范区、生态文明建设排头兵、面向南亚东南亚辐射中心，加快推进滇中城市经济圈一体化发展、打造昆玉红旅游文化产业经济带，有利于我市完善综合交通、提速城乡建设、优化产业布局、完善市场体系、带动投资需求、扩大发展空间。一定要加强政策研究，积极主动应对，顺时而谋、趁势而上，牢牢把握加快发展主动权。我们必须深刻认识、准确把握新常态下经济发展的着力点。要向深化改革要动力、向结构调整要助力、向民生改善要潜力，把抓产业、扩内需作为加快经济发展的重要着力点。围绕把玉溪建成全省产业转型升级先行区、新兴产业发展聚集区、重要高端旅游休闲度假区和重要内陆港的产业发展定位，巩固提升卷烟及配套、矿冶、高原特色农业三个支柱产业，着力培育旅游文化、装备制造、生物医药及食品、现代物流四个新的支柱产业，加快发展信息、新能源新材料及节能环保两个新兴产业。依靠深化投融资体制改革，优化财政支出结构，创新财政资金使用方式，引导社会资金投向重大基础设施和重点产业建设。依靠培育新兴业态激活消费潜力，促进消费结构升级。依靠深化行政审批制度改革，促进大众创业万众创新。依靠推进新型城镇化，以棚户区改造、新农村建设等为重点统筹城乡建设。

　　新常态下，玉溪经济发展、结构调整有较大空间和回旋余地。我们一定要提振发展信心，充分利用交通区位优越、生态环境良好、产业基础扎实、劳动力素质较高等优势，抓住关键，精准发力，扎扎实实抓好以下九项重点工作：

　　第一，发挥投资关键作用，进一步夯实发展后劲

　　坚持把增投资作为拉动经济增长的第一动力，优化投资结构，明确投资重点，确保规模以上固定资产投资达655亿元以上。

　　强化重点领域投资。设立产业发展引导基金，努力增加生产性项目特别是工业项目投资。突出综合交通、农田水利、能源信息等投资领域，发挥财政资金放大效益，撬动金融资本、社会资金有效投入。配合做好玉磨铁路征地拆迁和新临、弥玉高速公路前期工作，推进晋红、呈澄、石红高速公路建设，开工建设江通高速和新元一级公路，建设农村公路1000公里。改造中低产田地17.8万亩，实施烟叶生产基础设施项目1.7万件，配合做好滇中引水工程，推进元江鲁布、易门苗茂中型水库建设，开工建设元江小拉史、华宁核桃冲等6件小(一)型水库，完成新平马鞍山等3件小(一)型水源工程，病险水库除险加固58件，建成“爱心水窖”6000口，修复农村水毁工程。推进戛洒江一级、雨果等电站建设，抓好4个风电场和2个光伏发电项目建设，加快500千伏宁州变二期和2个220千伏、9个110千伏输变电工程建设，确保10个建成投产。

　　强化投资要素保障。抓住土地利用总体规划滚动修编机遇，加快“多规合一”步伐，建立土地指标分配与实际利用考核评价机制，加大土地储备力度，清查处置批而未用土地，盘活闲置土地，把有限的土地指标向积极性高、大项目好项目多并能及时落地的县区倾斜。鼓励民间资本发起设立产业投资基金和股权投资基金，支持民营资本参与重点领域投资，确保民间投资比重提高5个百分点以上。积极主动做好项目申报和协调汇报，争取上级资金增长10%以上。大力发展民生金融，鼓励金融机构创新金融产品，新增贷款80亿元。

　　强化项目推进责任落实。全面落实“七位一体”工作机制，建立季度综合调度制度，研究解决项目建设中的困难问题。强化重大项目推进责任，倒逼目标，倒排工期，倒查责任，坚决纠正业务部门之间互为前置、推诿扯皮、效率低下等行为。突出抓好省级“三个一百”和市级119个投资亿元以上重大项目建设，确保续建项目投资完成率80%、新上项目开工率70%以上。

　　第二，大力发展现代农业，促进农业增效农民增收

　　认真贯彻中央1号文件精神，全面实施高原特色农业发展规划，推动农业生产转方式、调结构、增效益、促增收，确保增加值增长6.5%以上。

　　发展特色优势产业。稳定粮食生产，加强仓储设施建设，确保粮食安全。种植烤烟54.1万亩、收购烟叶157.1万担，加强烟叶收购价格放开后的扶持政策研究，保障烟农利益。扶持发展蔬菜、花卉、林果等优势产业，新增核桃33.5万亩、水果2万亩、竹子1万亩。强化重大动物疫病防控，建设生猪标准化规模养殖场28个，扶持循环养殖示范场10个，加快畜牧业可持续发展，确保产值增长10%以上。

　　转变农业发展方式。落实龙头企业、农业庄园、家庭农场扶持政策，培育新型经营主体，推进8个省级精品庄园、50个家庭示范农场建设，做大“褚橙”、“猫哆哩”，做强“云菜”、“源天生物”等企业，新增龙头企业5户、农民专业合作组织40个。集中资金、技术、土地等生产要素，发展多种形式适度规模经营。新增土地流转面积3万亩。创新农村金融服务，维持县级农村信用社法人地位，鼓励发展村镇银行、小微金融。创新农业科技服务，抓好种苗繁育、技术配套和示范推广，创建国家农业科技园区。创新农产品营销方式，支持阿里巴巴搞好农村电子商务试点，建设特色中国玉溪馆。制定实施土壤污染治理年度计划，强化水旱轮作、配方施肥、降低复种指数等措施，大力发展资源节约、环境友好农业。启动可追溯体系建设试点，加强农产品质量安全监管。

　　拓宽农民增收渠道。大力发展农村二三产业，促进三次产业融合互动，培训农村劳动力2.25万人、转移1.55万人，鼓励就近就地兼业就业。全面落实粮食直补、农资综合直补、良种补贴、购机补贴、草原生态保护补助奖励等各项政策，扩大政策性农作物保险试点。实施精准扶贫，抓好4个整乡推进项目，完成30个自然村整村推进扶贫任务，加大产业扶贫、信贷扶贫力度，确保5万农村贫困人口脱贫。

　　第三，转变工业发展方式，推动产业提质增效

　　稳住存量、扩大增量，强化创新、做实园区，推动存量企业提档升级，引进重大项目扩能提质，确保工业增加值增长9.5%以上。

　　推进重点产业发展。全力为红塔集团提供优质原料，配合搞好打叶复烤搬迁技改、存储仓库等项目建设，支持卷烟产品结构调整，稳定生产销售规模;争取合和公司扩大玉溪投资，鼓励卷烟配套企业外引内联、加快发展。加强矿冶业转型升级政策措施研究，落实扩产促销、电价、税收等优惠政策，支持重点钢铁企业稳定生产、调整产品结构、延伸产业链。争取汽车产业布局玉溪，大力发展数控、风电、汽车配件等高端装备制造业。支持重点企业加快生物疫苗、化学制剂、中成药物等新产品开发，推动茶叶、菌类、果蔬、粮油等精深加工，促进生物医药及食品产业上规模、上档次、增效益。支持太阳能、风能、水能开发，加快锂电池、LED产业化，搞好再生资源回收利用，加快发展新能源新材料及节能环保产业。

　　加强工业园区建设。建立产业布局市级协调机制，每个园区确定2至3个主导产业，抓好水、电、路、气、通信等基础设施建设与城镇规划衔接配套，推进公共服务社会化，加快产城融合。推进园区实体化改革，赋予省级园区部分市级、县级经济社会管理权限，推行“2号公章制”，试行“跟章制”，消除体制机制障碍。创新融资方式，通过引进战略合作伙伴以PPP模式、争取省级发债等方式多渠道筹集资金，搞好土地收储、基础设施建设。主动融入滇中产业新区建设，加快高新区九龙片区、南片区产业转型升级和龙泉片区规划建设，推进大化园区和其他园区发展。落实园区属地政府招商引资责任，强化园区绩效考核，推进30万吨合金无缝钢管、1万台数控机床、韵雅黄腐酸、达利食品二期、30亿个食品包装盒等项目建设，确保投资5000万元以上重大项目30个开工、30个竣工，园区工业增加值增长10%以上。

　　大力发展民营经济。认真落实支持民营经济、小微企业发展的各项政策措施，确保民营经济增加值增长13%以上。加强银政银企合作，构建金融服务实体经济长效机制，鼓励企业利用前海股权交易中心挂牌融资，推进与上海股权交易所合作，年内2户以上企业实现新三板挂牌。制定政策措施，鼓励企业发展电子商务，促进互联网与实体产业跨界融合。建设微总部经济园区，实施中小企业成长工程和“两个10万元”微型企业创业培育工程，年内新增销售收入超50亿元企业2户、超10亿元企业4户，3户进入大企业行列、20户进入规模以上企业。突出特色，差异发展，促进县域经济做大做强。

　　强化创新驱动。落实加快实施创新驱动战略建设创新型玉溪的决定，实施“七大工程”，创建国家创新型试点城市。执行企业研发费用加计扣除等普惠性政策，支持企业建立研发机构、开发新技术新产品，新增院士工作站2个、重点实验室和工程技术中心7个。支持企业技术改造，认定高新技术企业7户、创新型企业3户。加强知识产权保护，专利申请量和授权量增长10%以上。建立专项基金，加强创新人才、职业经理、学科带头人、技师等重点人才的培养引进使用，创造人才红利。

　　第四，努力扩大城乡消费，加快第三产业发展

　　发挥消费基础作用，大力发展生产性、生活性服务业，创新经营业态，挖掘消费潜力，促进大众消费，推动消费升级，确保第三产业增加值增长12%以上。

　　着力发展壮大旅游文化产业。认真落实昆玉红旅游文化产业经济带建设玉溪行动计划，制定实施培育旅游产业新支柱行动方案，推动旅游与生态、文化、城乡建设融合发展。抓好澄江寒武纪乐园、仙湖锦绣、仙湖时光栈道等重大项目建设，加快抚仙湖—星云湖生态建设与旅游改革发展综合试验区发展。加强优质资源管控，引导社会资本参与建设开发，打造“三湖”休闲度假、哀牢山—红河谷民族生态、易门—红塔—华宁特色旅游3条旅游线路。积极发展乡村旅游。推进智慧旅游工程，建立旅游联合促销联盟，扩大客源市场，确保旅游总收入增长16%以上。

　　加快服务业提质增效。着力推进信息惠民、信息消费、宽带乡村试点城市建设，加快华为玉溪云计算数据中心、智慧玉溪等项目建设，积极发展信息服务业。大力发展现代物流业，搞好物流园区规划布局，加快通力汽运、润特物流等项目建设，引进知名物流企业，打造国际贸易流通全省重要内陆港。推进聂耳美食文化城建设，开发地方特色菜品，打造餐饮品牌。大力发展金融、保险、商贸等服务业，积极培育律师、评估、咨询等中介组织。

　　努力扩大城乡消费。实施养老健康家政、信息、旅游、住房、绿色、教育文化体育6大领域消费工程，培育消费增长点。认真落实不动产登记条例和住房贷款优惠政策，促进商品房去库存化;加强公租房出租出售政策研究，提高入住率;加快美丽家园建设、农村危房改造，努力扩大城乡住房消费。抓好汽车、家电等大宗消费，培育教育、文化、节能环保等消费热点。加快得胜商业中心和3个农产品批发交易市场建设，完成11个乡镇集贸市场、10个便民农超建设任务，进一步改善消费环境。

　　第五，深化改革开放，增强发展活力动力

　　坚持把改革开放作为加速发展的动力源泉，切实推进重点领域和关键环节改革，着力提高对外开放水平，向改革要红利，向开放要效益，激发市场和社会活力。

　　深化重点领域改革。深化行政审批制度改革，抓好行政审批事项承接、下放和监管。完成新一轮政府机构改革任务。深化农村产权制度改革，推进县乡土地流转服务中心和综合性农村产权交易平台建设，全面推进农村土地承包经营权确权登记颁证工作、完成100万亩颁证，推动农村土地承包经营权、宅基地使用权、房屋所有权“三权三证”抵押融资工作，推行土地征收补偿费作价入股模式。进一步深化集体林权制度改革，推进水务、供销、粮食等改革。完成6个省级工业园区实体化改革任务。推进财税金融改革，深化预算管理制度改革，完成“营改增”任务，把市商业银行由地方性银行转型发展为区域性商业银行。深化投融资平台改革，整合业务、配实资源、理顺体制、市场运营，创新投融资方式，促进投融资平台做强做实。重视和加强地方政府性债务管理，妥善处理存量债务和在建项目后续融资，规范举债融资行为，防范化解财政金融风险。全面深化教育综合改革，抓好县级公立医院改革，开展新农合支付方式改革试点工作。完成殡葬制度改革三年目标任务。开展机关事业单位养老保险制度改革，统筹搞好生态文明、科技、文化等体制改革。

　　加大招商引资力度。坚持政策一宽再宽、利益一让再让、服务一改再改、环境一造再造，完善招商引资促进政策，建立招商引资项目开发、准入和退出机制。推广易门县主要领导高位推动、突破机制障碍、全程高效服务、全民合力招商等成功经验，强化县区、部门责任落实和考核问责，确保市外国内到位资金增长18%以上、实际利用外资增长10%以上，力争形成投资500亿元以上。突出招大引强引智，紧盯行业领军企业实施精准招商、驻点招商，组织重大招商引资活动实施产业招商、园区招商、以商招商，提高招商引资实效。建立重大项目行政许可代理服务制度，加强项目合同管理，搞好协调服务和落地督办，提高合同履约率、项目开工率和资金到位率。强化项目开发，年内储备投资超5亿元招商引资项目100个以上。

　　努力扩大对外贸易。制定支持外经外贸发展政策，引导企业积极稳妥“走出去”投资兴业，推动外经外贸联动发展。实施更加积极的进出口政策，搞好贸易便利化服务，支持重点企业拓展国际市场，大力发展加工贸易，扩大果蔬和高附加值产品出口，确保外贸进出口总额增长18%以上。

　　第六，加快新型城镇化进程，促进城乡一体化发展

　　完成新型城镇化规划编制，全面落实推进新型城镇化发展意见，以历史耐心推进以人为核心的新型城镇化。

　　主动融入滇中城市经济圈一体化发展。制定完善玉溪市加快滇中城市经济圈一体化发展的政策措施，推动基础设施、产业发展、市场体系、基本公共服务和社会管理、城乡建设、生态环保一体化，加快昆玉一体化进程，率先在滇中城市经济圈一体化发展上取得突破。科学安排山、水、田、路、产、城，完善中心城区城市功能，积极推进江川、澄江撤县设区和通海撤县设市工作，构建沿湖4县半小时经济圈，推进红塔区、江川、澄江、通海、华宁、峨山一体化发展，打造“三湖”生态城市群。打好综合交通建设攻坚战，构建以高速公路、铁路为主骨架的现代综合交通运输体系，完成昆玉、玉元高速清水河至高仓段亮化工程，启动昆明—澄江—江川—红塔区—通海城际铁路建设前期研究，加快与其他州市的互联互通。加强与省和滇中州市的沟通对接，全力争取政策、项目、资金支持，实现产业分工互补协作、市场建设互融互通、生态建设同防同治。

　　加快中心城区规划建设。围绕“一城四点”空间布局，统筹新区开发和老城改造，统筹园区开发和城市建设，统筹地上地下，建设全省区域性中心城市。完成道路竖向、停车系统和综合交通等专项规划编制，加强规划管理信息平台建设。争取全国海绵城市建设试点，完成1∶500地形图及地下管线测绘，规范地下综合管网建设。启动临岸三千城、规划展览馆建设，加快新天地、红星国际广场建设和红龙路改扩建，完成梅园立交拆除重建、排水管网改扩建二期等项目建设。优化公交路网布局，推进公交始末站、城南城北客运站、公交综合大楼项目。建设燃气管道32公里，新增居民用户2万户。搞好集体低效闲置和拆临拆违地块综合开发利用。巩固国家卫生城市、园林城市成果，争创全国智慧城市。

　　着力推进县城集镇乡村建设。完成美丽玉溪行动规划编制和通海、易门、峨山县城总体规划修编，完善提升村庄规划。完成红塔区、易门县“四规合一”试点工作，合理确定城镇规模、开发边界、开发强度和保护空间，鼓励社会资本参与开发建设，以城聚产、以产兴城、产城联动。坚持政府主导、市场运作，启动实施6个县区9个棚户区改造项目，力争完成投资100亿元以上。扎实开展“百村示范千村整治”行动，整合乡村建设资金，启动12个集镇“一水两污”项目，完成18个集镇重点基础设施建设，建成40个示范村，整治400个重点村。深化户籍制度改革，完善配套措施，引导农业转移人口市民化。

　　提高建设管理水平。加强规划管理，严格规划执行，坚决整治乱占乱建乱加层等行为。严格执行城市管理条例及配套办法，深入推进城市精细化管理，加强城市综合执法，着力解决交通拥堵、占道经营、车辆乱停乱放、出租车违规经营等突出问题。加强城市环境卫生管理，集中整治背街小巷、城中村和城郊结合部脏乱差问题。加强农村建房管理，完善建房审批程序，提高审批效率，满足群众合理需求。

　　第七，加强环境保护治理，推进绿色玉溪建设

　　落实绿色玉溪行动计划，实施森林玉溪、蓝天玉溪、碧水玉溪、园林玉溪、绿色产业、绿色文化6大工程，最大限度增绿添绿，最严举措低碳发展，最硬措施保护生态，争创中国人居环境奖，争当全省生态文明建设排头兵。

　　加大“三湖两库”保护治理力度。严格落实“三湖”保护条例和抚仙湖保护管理实施办法，争取修订《云南省抚仙湖保护条例》。坚决贯彻市委“五个坚定不移”工作要求，强化工程和非工程措施落实，确保“十二五”规划项目全面完工，抚仙湖总体水质保持I类、星云湖和杞麓湖水质有所好转。着力实施“四退三还”，加快抚仙湖北岸生态湿地工程建设，建成北岸生态调蓄带，稳妥推进中央、省属12家单位资产退出一级保护区工作。按质按量完成东片区暨“三湖”生态保护水资源配置应急工程，制定配套措施，确保正常运行。完成村庄环境整治、入湖河道治理等28个项目建设。完善沿湖4县生态建设目标任务考核办法，制定差异化扶持政策，争取澄江、江川、华宁3县纳入国家重点生态功能区转移支付范围，鼓励引导沿湖4县转型发展。加大结构调整力度，推广核桃、蓝莓、荷藕种植，逐步取缔径流区规模养殖。划定星云湖、杞麓湖流域生态红线，搞好星云湖环湖截污。推广沿湖环境卫生网格化、市场化运作模式，巩固提升入湖河道管护成果。加强东风水库、飞井海水库环境综合整治。

　　加大生态建设力度。实施绿化造林、退耕还林、石漠化综合治理等工程，完成营造林37.5万亩，退耕还林3万亩，加强生物多样性保护，严防森林火灾和病虫害发生。开展全国水生态文明城市试点工作，抓好澄江、华宁节水减排示范项目建设，搞好14个集中饮用水源地环境综合整治。加强元江、曲江等河流水污染综合防治，治理水土流失190平方公里。开发整理土地，新增耕地1.5万亩，实现耕地占补平衡。完善地质、气象、地震等灾害防御体系，完成地质灾害避让搬迁任务。

　　加大环境监管力度。化解过剩产能，淘汰43万吨钢铁、76万吨水泥、2万吨铁合金落后产能，严格新建项目能耗达标管理，加强重点企业能耗、污染源在线监测，实施115个减排项目，深入开展国家循环经济试点，确保“十二五”节能减排目标任务圆满完成。加强施工场地和园区环境综合整治，开展机动车环保检测，淘汰黄标车和老旧车辆，做好中心城区和县城PM2.5监测发布。加强交通运输、建筑工地、工矿企业等噪声监管，开展宁静社区示范建设。探索环境污染第三方治理，严格执行重点项目建设环评和“三同时”制度，严厉查处违法排污、破坏生态行为。

　　第八，全力保障和改善民生，确保社会和谐稳定

　　更加注重保障基本民生，更加关注低收入群体生活，健全完善社会托底政策，推进基本公共服务均等化，促进社会公平正义，让发展成果更多更公平惠及全市人民。

　　全力做好社会保障。落实促进就业创业政策，实施高校毕业生就业促进计划和创业引领计划，加快大学生创业园建设，促进就业困难人员和退役军人就业创业，确保城镇新增就业2.2万人。扩大社会保险覆盖面，健全完善工伤、生育保险制度，做好失业保险扩面和被征地农民养老保险工作，提高城镇居民医疗保险、新农合筹资标准。加强保障性住房建设，实施城市棚户区改造1万户、农村危房改造1.28万户。大力发展养老服务，支持社会力量举办养老机构，改扩建农村乡镇敬老院6个，建成社区居家养老服务中心40个、农村幸福院70个。提高城乡低保补助标准，完善价格临时补贴与物价上涨联动机制，推进新型社会救助体系建设，搞好救灾救济，保障低收入群体基本生活。推进移民新村建设，做好化念移民后期扶持工作。健全殡葬服务体系，确保火化区火化率和公墓安葬率均达100%。

　　大力发展社会事业。推进美丽100校园行动计划暨校安工程建设，启动“全面改薄”工程和第二期学前教育三年行动计划，加快教育信息化建设，改善办学条件;加强师德师风和业务能力建设，提高教育教学水平;扶持发展学前教育，均衡发展义务教育，扩大高中办学规模，统筹办好高等教育、职业教育、特殊教育。继续推进县乡村医疗卫生服务一体化管理，提高基层医疗卫生机构服务能力和救治水平。完善公共卫生服务体系，强化食品药品安全监管。加快市医院改扩建、市儿童医院、市急救中心、西南国际医院暨健康产业园等重大项目建设。落实“单独两孩”政策，创建流动人口卫生和计生基本公共服务均等化示范市。广泛开展全民健身活动。重视妇女儿童、残疾人、红十字等事业，加强外事侨务、保密、档案、史志等工作。

　　着力打造文化玉溪。落实加快文化玉溪建设实施方案，培育和践行社会主义核心价值观，做好省级社会信用体系建设试点工作。推进澄江化石地世界自然遗产保护立法，发挥世界遗产品牌效益，加快化石博物馆建设和文化创意衍生品研发，挖掘提升古滇文化、铜文化、陶瓷文化、民族文化特色内涵，打造“一地四乡”文化名片。实施文化惠民工程，加强文化基础设施建设，抓好中央节目地面数字无线覆盖和6座高山无线发射台站建设。实施文艺精品、文化名家工程，繁荣文艺创作。加快玉溪瓷文化创意产业园建设，培育陶瓷、青铜器加工、出版等文化产业。办好第四届聂耳音乐合唱周系列活动，创建中国楹联文化城市，力争通海列为国家历史文化名城。

　　努力创建平安玉溪。执行“四五”依法治市规划，完成“六五”普法任务。创新社会管理方式，加强社区基础设施建设，推进基层群众自治，提高社会治理能力。认真贯彻民族区域自治法，加强民族团结进步边疆繁荣稳定示范区建设，持续抓好新平示范县、盘溪示范镇和40个示范村创建工作，依法管理宗教事务。强化属地管理责任，深入开展领导干部接访下访，建立完善视频接访和联合接访制度。完成城市报警和监控系统建设，加强反恐维稳工作，打好禁毒防艾人民战争，严厉打击各类违法犯罪，争创全国“长安杯”、平安云南建设示范区。强化安全生产党政同责，建立大检查长效机制，打好重点行业领域攻坚战，加强烟花爆竹行业监管，禁止铁水道路运输，严防重特大事故发生。加强国防动员、民兵预备役和人民防空工作，完成武警支队迁建，争创全国双拥模范城。

　　继续办好10件惠民实事。

　　第九，精心编制“十三五”规划，科学谋划未来发展

　　深化市情认识，找准发展定位，抓好重点专项规划、区域规划编制和重大政策、重点课题研究，加强与国家、省“十三五”规划编制的衔接，精心谋划玉溪“十三五”经济社会发展。紧密结合中央和省的政策导向，立足当前、谋划长远，搞好项目设计论证、筛选申报，争取产业发展、综合交通、农田水利、生态环保、社会民生等一批重大项目列入国家和省的规划盘子。

　　三、以法治政府为重点加强政府自身建设

　　正确处理政府与市场、政府与社会的关系，切实把政府工作重点转移到营造环境、提供服务、维护公平正义上来，努力建设法治、高效、廉洁、阳光政府。

　　第一，全面加快法治政府建设。制定法治政府建设指标体系，推进政府机构、职能、权限、程序、责任法定化，编制、公布政府及其工作部门权责清单，推进决策、执行、管理、服务、结果“五公开”，把权力关进制度的笼子，让权力在阳光下运行。维护市委统揽全局、协调各方的核心领导地位，自觉接受市人大法律监督、工作监督和市政协民主监督，认真办好人大代表建议和政协委员提案。健全依法决策机制，完善重大行政决策公众参与、专家论证、风险评估、合法性审查、集体讨论决定等制度，修订行政机关法定代表人行政诉讼出庭应诉制度，出台加强政府法律顾问工作的意见，执行重大决策终身责任追究制度及倒查制度，发挥决策咨询机构作用。深化行政执法体制改革，健全行政执法与刑事司法衔接机制，加强重点领域综合执法，建立健全行政裁量权基准制度，充实基层执法力量，强化行政执法监督，严格规范公正文明执法。

　　第二，提高政府服务效率水平。巩固党的群众路线教育实践活动成果，认真执行中央八项规定，坚决防止“四风”反弹回潮，不当“四拍”领导、不做“四皮”干部，发扬“钉钉子”精神，狠抓工作落实，扎扎实实服务基层、服务群众、服务企业。转变文风会风，开短会、讲短话，出实招、求实效，把全部心思和精力集中到干事创业、推动发展上。落实干部直接联系群众制度，推行随机调研，矛盾在一线化解，工作在一线推进。进一步简化行政审批程序，落实审批授权、并联审批、重大项目代办、三级联动审批四项制度，与省同步运行网上政务服务大厅。落实敬业有功、怠业必惩实施方案，强化督促检查、行政问责，着力整治不作为、乱作为、慢作为和“三难四多”问题，打通决策部署“最先一公里”和政策落实“最后一公里”。

　　第三，切实做到廉洁从政。牢固树立改革、发展、稳定、反腐“四位一体”工作理念，履行党风廉政建设“一岗双责”，健全完善惩治和预防腐败体系，严格执行“三重一大”、任期经济责任审计等制度，重点加强行政审批、招投标、公共资源交易、财政预算、国有资产等领域监管。深入开展“三严三实”和“忠诚干净担当”专题教育，勤用“四盆水”，做到“六个不能”，当好“六个表率”。严格执行新预算法，实行全口径预算，杜绝超预算、无预算安排支出，整治私设“小金库”、暗藏“私房钱”。厉行节约反对浪费，完成公务用车改革，建立机关事务管理制度和标准，严控“三公”经费，压缩一般性支出，提高财政资金使用效益。严肃查处违法违纪案件，以为政清廉取信于民，以秉公用权赢得民心。

　　各位代表，面对经济发展新常态，加快发展的要求更高、任务更重。让我们在市委的坚强领导下，更加紧密地团结在以同志为的党中央周围，凝心聚力、奋发有为，干在实处、走在前列，为全面建成小康社会做出新的更大的贡献!
